# Double-bowl state in photonic Dirac nodal line semimetal

**DOI:** 10.1038/s41377-021-00614-6

**Published:** 2021-08-20

**Authors:** Mengying Hu, Ye Zhang, Xi Jiang, Tong Qiao, Qiang Wang, Shining Zhu, Meng Xiao, Hui Liu

**Affiliations:** 1 National Laboratory of Solid State Microstructures, School of Physics, Collaborative Innovation Center of Advanced Microstructures, Nanjing University, 210093 Nanjing, China; 2 Division of Physics and Applied Physics, School of Physical and Mathematical Sciences, Nanyang Technological University, Singapore, 637371 Singapore; 3 Key Laboratory of Artificial Micro- and Nano-structures of Ministry of Education and School of Physics and Technology, Wuhan University, 430072 Wuhan, China

**Keywords:** Optics and photonics, Nanophotonics and plasmonics

## Abstract

The past decade has seen a proliferation of topological materials for both insulators and semimetals in electronic systems and classical waves. Topological semimetals exhibit topologically protected band degeneracies, such as nodal points and nodal lines. Dirac nodal line semimetals (DNLS), which own four-fold line degeneracy, have drawn particular attention. DNLSs have been studied in electronic systems but there is no photonic DNLS. Here in this work, we provide a new mechanism, which is unique for photonic systems to investigate a stringent photonic DNLS. When truncated, the photonic DNLS exhibits double-bowl states (DBS), which comprise two sets of perpendicularly polarized surface states. In sharp contrast to nondegenerate surface states in other photonic systems, here the two sets of surface states are almost degenerate over the whole-spectrum range. The DBS and the bulk Dirac nodal ring (DNR) dispersion along the relevant directions, are experimentally resolved.

## Introduction

Discovering new topological phases of matter is of significant importance for both fundamental physics and materials science^1–7^. Theory of symmetry indicators is successful in identifying electronic topological materials^8^. With mature algorithm developed, extensive efforts have been taken to diagnose topological characters of electronic materials in the crystal structure database exhaustively^9–11^. The topological classification of the photonic systems was originally thought to be a trivial extension of the electronic counterpart and described by spinless space groups. However, detailed analyses reveal that photonic systems are distinct from the electronic counterparts, and connectivity at zero frequency in dielectric materials and hidden symmetry enforced nexus points are latter found to be unique to photonic systems^12,13^. Here in this work, we provide a stringent photonic realization of Dirac nodal line semimetal (DNLS), which is not a spinless version of the electronic DNLS. More intriguingly, such a photonic DNLS exhibits perpendicularly polarized double-bowl surface states (DBS), which are degenerately pinned at the bowl center and bowl edge and are almost degenerate over the entire spectrum range. This is in sharp contrast to other photonic systems, where the two perpendicularly polarized states are in general nondegenerate.

DNLSs^14–18^ and three-dimensional (3D) Dirac semimetals^19^ with four-fold band degeneracy stand as important members of the topological semimetal family^14–24^. They exhibit various unique properties such as giant diamagnetism^25^, flat Landau levels^26^, and long-range Coulomb interaction^27^, among others^28^. In addition, they are neighbors to many novel topological phases and thus serve as ideal platforms for investigating topological phase transitions^19^. Three-dimensional Dirac semimetals have been observed in both electronic systems and classical waves^19,29–31^. In electronic systems, DNLSs are possible in the absence of spin-orbital couplings^16–18^. Meanwhile, they can also be protected by nonsymmorphic symmetries in the presence of spin-orbital couplings^14,15^. However, there is NO photonic DNLS in all previous works.

The effective Hamiltonian of a simple Dirac nodal ring (DNR) degeneracy of a DNLS in the *x*–*y* plane can be written:

1 $$H = \left[ {\left( {q_\rho - q_0} \right)\sigma _x + q_z\sigma _z} \right]\tau _0$$

where $\tau _0$ is the 2 × 2 identity matrix, $\sigma _x$ and $\sigma _z$ are Pauli matrixes, $q_0$ is the radius of the nodal ring, and $q_\rho$ and $q_z$ represent the wave vector along the radial and *z* directions, respectively. Such a Hamiltonian possesses a four-fold ring degeneracy along the polar angle $\hat \varphi$ direction at $q_\rho = q_0$ and $q_z = 0$. According to convention, we use a *σ* matrix to represent the band index and a $\tau$ matrix to represent the (pseudo-) spin index. A two-fold Weyl nodal line degeneracy can be easily constructed by intersecting two bands with different representations of a certain symmetry such as the mirror symmetry, PT symmetry, or glide symmetry^14^. However, the extension of the two-fold Weyl line degeneracy to a four-fold degeneracy DNR is not an easy task. Currently, all the nodal line semimetals in classical waves have been Weyl nodal line semimetals with two-fold band degeneracy^24,32–34^, and there has been no DNLS in classical waves. For light, the pseudo-spin freedom is interpreted as the polarizations. In principle, the optical responses of different polarizations can be tuned to be identical by setting $\varepsilon = \mu$; however, this is experimentally impractical. Additionally, the lattice symmetries exhibit higher dimensional representations only at highly symmetry points. The nonsymmorphic symmetries that protect the electronic DNR do not work for light due to the inherent distinction between fermions (electron) and bosons (photon); i.e., the time reversal operator squares to –1 for fermions, whereas it is +1 for bosons. Thus, to construct an optical DNLS, a brand new mechanism is here established in order to ensure that the coefficients in front of all τ_x_, τ_y_ and τ_z_ matrixes in the Hamiltonian vanish over a certain parameter range.

## Results

**Fig. 1:**
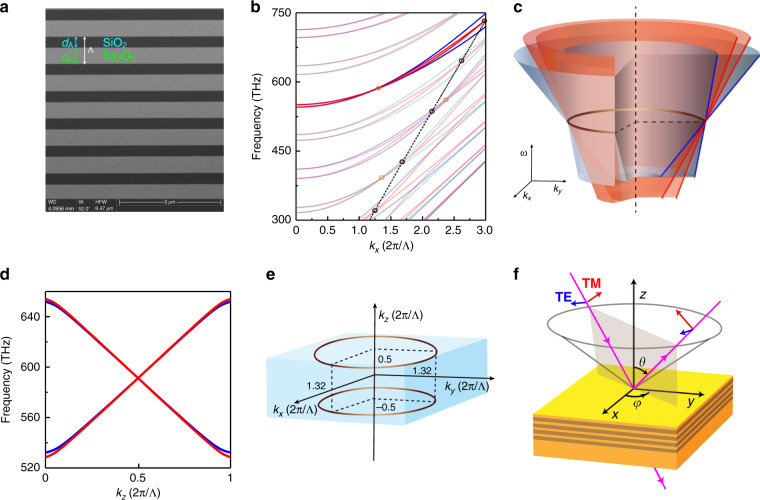
Photonic DNLS. **a** SEM image of the PC. **b** Dispersion along the $k_x$ direction at $k_z = 0$ (green and magenta) and $k_z = \pi /{\Lambda}$ (red and blue). The blue and magenta lines represent TE polarization, and the red and green lines represent the TM polarization. The bands forming the DNR, which is the focus in our study, are highlighted, while the other bands are partially transparent. The dashed line indicates the locus of the Brewster angles, where the TM gaps close. The gap closing points for both polarizations (at the Brewster angles) are encircled in orange (black). **c** Sketch of the in-plane dispersion around the DNR (golden). The DNR is four-fold degenerate, consisting of two sets of type-II Weyl nodal rings of TE (blue) and TM (red) polarizations. **d** Dispersions along the $k_z$ direction around the four-fold degeneracy point for TE (blue) and TM (red) polarizations. **e** The position of the DNR in the reciprocal space. **f** The experimental setup. We employ SiO_2_ (Ta_2_O_5_) for layer A (B), with a thickness of *d*_A _= 388 nm (*d*_B _= 597 nm). The refractive index of the SiO_2_ (Ta_2_O_5_) is around 1.48 (2.25) with slight dispersion in the visible regime (see Supplementary Data I).

Our system is an AB layered photonic crystal (PC) with SiO_2_ ($\varepsilon _{{{\mathrm{A}}}} \approx 2.18$) and $d_{{{\mathrm{A}}}} = 388\;{{{\mathrm{nm}}}}$ for layer A, and Ta_2_O_5_ ($\varepsilon _{{{\mathrm{B}}}} \approx 5.06$) and $d_{{{\mathrm{B}}}} = 597\;{{{\mathrm{nm}}}}$ for layer B. This structure can be fabricated with the e-beam evaporation technique. An SEM picture of our sample is shown in Fig. 1a. Simple as it is, this structure is perceived as a photonic DNLS. The band degeneracies of this system are only found at $k_z = 0$ or $k_z = \pi /{\Lambda}$, (see ref. ^35^ and Supplementary information Sec. I), where ${\Lambda} = d_{{{\mathrm{A}}}} + d_{{{\mathrm{B}}}}$ is the unit cell length and $k_z$ is the Bloch wave vector perpendicular to the layers. Thereby, to identify the band degeneracies of this system, it is only necessary to plot the band edge states at at $k_z = 0$ and $k_z = \pi /{\Lambda}$ as a function of $k_x$ while keeping $k_y = 0$, as shown in Fig. 1b. The pseudo-spin freedom shown in the figure consists of the transverse-electric (TE) and transverse-magnetic (TM) modes, featured by the electric and magnetic fields only in the in-plane directions, respectively. Basically, there are two types of band degeneracies, marked in Fig. 1b with black circles and orange circles. The black circle degeneracies are due to the Brewster angle where the impedances of the two layers match, which is only possible for the TM modes (the red and green bands). They lie along an essentially straight black dashed line since the material dispersions are small in the frequency range of interest (index variation <1.9% for SiO_2_ and <6.4% for Ta_2_O_5_; see Supplementary Data I for measured refractive index).

In addition to the degeneracy induced by the Brewster angle, there is another type of band degeneracies for both the TE and TM modes, marked with orange circles in Fig. 1b. Interestingly, the degeneracies of TE and TM modes occur at identical $k_x{{{\mathrm{s}}}}$. It is here proven that (see proof in Supplementary information Sec. I) such band degeneracies exist when

2 $$\tilde n_{{{\mathrm{A}}}}d_{{{\mathrm{A}}}}/\tilde n_{{{\mathrm{B}}}}d_{{{\mathrm{B}}}} = m_1/m_2 \in {\Bbb Q}$$

where $m_1,m_2 \in {\Bbb N}^ +$, and $\tilde n_i = \sqrt {\varepsilon _i\mu _i - k_x^2/k_0^2}$ ($i = {{{\mathrm{A}}}}\;{{{\mathrm{or}}}}\;{{{\mathrm{B}}}}$) with $k_0$ being the wave vector in a vacuum. Under such a condition, the $\left( {m_1 + m_2} \right)^{{{{\mathrm{th}}}}}$ band and the $\left( {m_1 + m_2 + 1} \right)^{{{{\mathrm{th}}}}}$ band cross at

3 $$f_{m_1 + m_2} = \left( {m_1 + m_2} \right)c/2\left( {\tilde n_{{{\mathrm{A}}}}d_{{{\mathrm{A}}}} + \tilde n_{{{\mathrm{B}}}}d_{{{\mathrm{B}}}}} \right)$$

where *c* is the speed of light in a vacuum. Equations (2) and (3) extend the relationship in ref. ^33^ at the normal direction to off-normal directions at finite $k_x$. We emphasize that Eqs. (2) and (3) work for both TE and TM polarizations and hence lead to a four-fold degeneracy. In addition, the existence of such band degeneracies is not accidental. It depends little on the material dispersions nor requires specific materials. Moreover, $k_x{{{\mathrm{s}}}}$ of the four-fold degeneracies can be simply controlled by varying $d_{{{\mathrm{A}}}}$ and $d_{{{\mathrm{B}}}}$ for the chosen materials (see more details in Supplementary information Sec. I).

We here focus on one of the four-fold degeneracies around 591 THz, as stressed in Fig. 1b. Considering the fact that our system is rotationally invariant, if there exists a degeneracy at $k_x = k_{\rho D}$ and $k_y = 0$, such a degeneracy ought to be extended to form a ring shape at $k_{\rho D} \equiv \sqrt {k_x^2 + k_y^2}$. Figure 1c sketches the in-plane band degeneracy around $k_{\rho D} = {{{\mathrm{1}}}}{{{\mathrm{.32}}}}\left( {{{{\mathrm{2}}}}\pi /{\Lambda}} \right)$, from which we can see that two linear TM (red) bands are sandwiched by two linear TE (blue) bands and all the bands are degenerated at the golden ring at $k_{\rho D}$. The dispersions of these four bands are all positive along the in-plane radial direction. Figure 1d shows the dispersion along the $k_z$ direction around this four-fold degeneracy. All the bands have linear dispersion away from the degenerate point. Thus, we evidence the existence of DNLS with nodal line degeneracy at $k_{\rho D} = {{{\mathrm{1}}}}{{{\mathrm{.32}}}}\left( {{{{\mathrm{2}}}}\pi /{\Lambda}} \right)$, $k_z = \pi /{\Lambda}$, and $f = 591\;{{{\mathrm{THz}}}}$, as sketched in Fig. 1e. Combined with the dispersions shown in Fig. 1b and d, one can conclude that the DNLS shown in Fig. 1 belongs to type II^36^. The ring degeneracy for each polarization in our system is protected by the intrinsic mirror symmetry of the AB layered structure. Meanwhile, the degeneracy between different polarizations is required by Eqs. (2) and (3). In contrast to the DNLS protected by the nonsymmorphic symmetries in electronic systems, in our system, the DNRs can be found at both $k_z = 0$ and $k_z = \pi /{\Lambda}$. (The DNR at $k_z = 0$ is provided in Supplementary information Sec. II.)

The experimental setup is depicted schematically in Fig. 1f, where we use yellow and brown to depict the layers made of SiO_2_ and Ta_2_O_5_, respectively. The number of unit cells of our PC is 12, which is not shown explicitly in Fig. 1f. Light is incident (magenta) with either the TE or TM polarization on the sample with the incident angle *θ*, which determines the parallel wave vector excitation. In our experiments, we cover *θ* from $0^ \circ$ to $60^ \circ$. The azimuth angle $\varphi$ can be flexibly tuned to verify the in-plane isotropy. The transmission and reflection spectra as functions of the frequency ($f$), *θ* and $\varphi$ are here collected and analyzed.

**Fig. 2:**
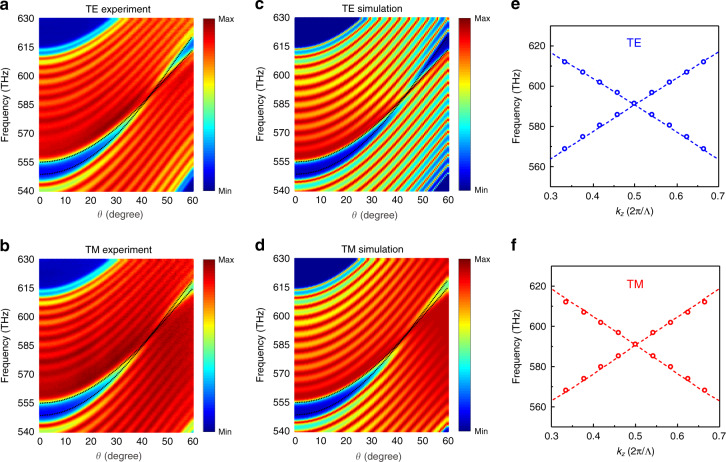
Experimental observation of photonic DNR. **a**, **b** Measured and **c**, **d** simulated transmission spectra for the PC sample as the incident angle varied from $0^{{{\mathrm{o}}}}$ to $60^{{{\mathrm{o}}}}$ for the TE and TM plane-wave excitations. The transmission spectra fill the region bounded by the bulk bands (black dashed lines) along the $k_x$ direction at $k_z = \pi /{\Lambda}$. **e**, **f** Extracted dispersions along $k_z$ from the Fabry-Perot interference pattern for **e** TE and **f** TM polarizations, in which the open circles are acquired from the transmission peaks. The dashed lines are theoretical results for reference. The dielectric and geometric parameters of the PC are the same as those shown in Fig. 1.

We perform angle-resolved transmission measurements on the PC sample to probe the dispersions of the DNLS, in which we start by setting $\varphi = 0^ \circ$ without loss of generality. The experimental results are given in Fig. 2a and b for the TE and TM polarizations, respectively, alongside with the corresponding simulation spectra in Fig. 2c and d for comparison. We then carry out measurements at other $\varphi$s (Supplementary information Sec. III), and the measurements are almost identical to those at $\varphi = 0^ \circ$, which thus fully confirms the in-plane isotropy of our sample. Notice that the numerical and experimental results are closely consistent with each other. Since the dispersions along the $k_z$ direction are monotonic (Supplementary information Sec. I), the transmission spectra fill the projected band region with the boundary given by the black dashed lines for the dispersion along $k_x$ at $k_z = \pi /{\Lambda}$. Hence, we obtain the band dispersions along the $k_x$ direction at $\varphi = 0^ \circ$, which linearly cross each other at $\theta = 44^{{{\mathrm{o}}}}$ and $f = 591\;{{{\mathrm{THz}}}}$ for both the TE and TM polarizations. The fringe pattern on the transmission spectra intrinsically stems from the Fabry-Perot interference of the Bloch modes, for which the peak frequencies satisfy:

4 $$Nk_z{\Lambda} = m\pi$$

In this expression, $N = 12$ is the number of unit cells of our sample, $k_z$ is the corresponding Bloch wave vector, and $m \in {\Bbb Z}$. By the aid of Eq. (4), we are capable to obtain the dispersion along $k_z$ at $k_x = k_{\rho D}$ and $\varphi = 0^ \circ$, as shown in Fig. 2e and f, in which the dashed lines are theoretical results and the open circles come from the peak data (details can be found in Supplementary information Sec. IV). Figure 2 indicates that the two bands cross each other linearly along both the $k_x$ and $k_z$ directions for either the TE or the TM polarization at the same degeneracy point. Considering that the band dispersions are identical for all the azimuth angles, this four-fold degeneracy point actually extends to a ring shape, i.e., DNR, in the $k_x - k_y$ plane. Consequently, we experimentally demonstrate the existence of photonic DNLS.

**Fig. 3:**
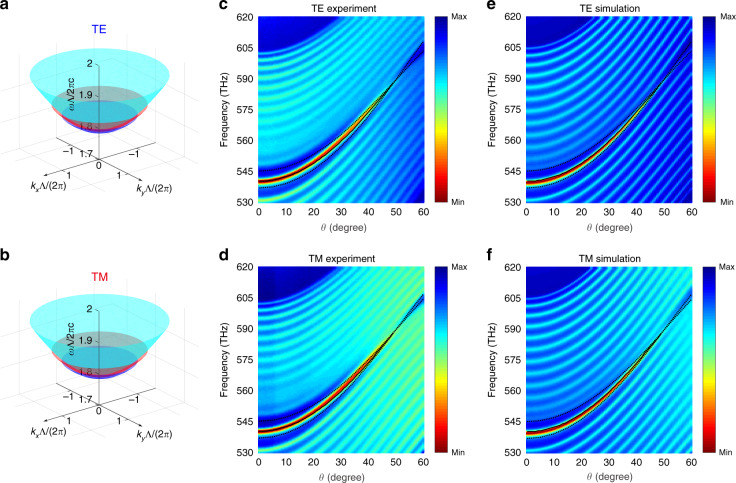
Observation of the DBS. **a**, **b** Bowl surface state dispersion for **a** TE and **b** TM polarizations, in which the cyan, red, and dark blue surfaces indicate cases in which $n_{{{\mathrm{A}}}}d_{{{\mathrm{A}}}}/n_{{{\mathrm{B}}}}d_{{{\mathrm{B}}}}$ equals 0.442, 0.422, and 0.415, respectively. $n_{{{\mathrm{A}}}}d_{{{\mathrm{A}}}} + n_{{{\mathrm{B}}}}d_{{{\mathrm{B}}}}$ remains constant in these simulations. In the experiment, $n_{{{\mathrm{A}}}}d_{{{\mathrm{A}}}}/n_{{{\mathrm{B}}}}d_{{{\mathrm{B}}}}$ of our PC is 0.442. **c**, **d** Measured and **e**, **f** simulated reflection spectra for the silver film/PC sample as the incident angle changes from $0^{{{\mathrm{o}}}}$ to $60^{{{\mathrm{o}}}}$ for TE and TM excitations. The black solid lines mark the numerically simulated bowl surface states dispersion, while the black dashed lines show the dispersion of the bulk PC bands. The PC shares identical dielectric properties with those shown in Fig. 2 but with slightly different thicknesses of *d*_A_ = 402 nm and *d*_B_ = 605 nm. The top layer of the truncated PC is layer B with $d_{{{\mathrm{B}}}}/2$. The thickness of the silver film on the top is 25 nm ($\pm 5\;{{{\mathrm{nm}}}}$).

In addition to the DNR, our system exhibits a new type of nearly degenerated surface states. To demonstrate this, we deposit a silver film with a thickness of 25 nm on the PC to confine light. Tamm-like surface states are formed between the silver film and PCs. Since we have a DNR, these Tamm-like surface states exist for both polarizations. It is worth noting that the Tamm-like surface can either be expanded by the DNR, or extended from the DNR to infinity, depending on the detail of the PC surface truncation. In our case, the PC is truncated with half of layer B on top, and with this setup, the composite system exhibits the surface states for both polarizations (The Tamm-like surface states that extended from the DNR to infinity are shown in the Supplementary information, Sec. V.). Figure 3a and b exhibit the surface states for three typical values of $n_{{{\mathrm{A}}}}d_{{{\mathrm{A}}}}/n_{{{\mathrm{B}}}}d_{{{\mathrm{B}}}}$ for the TE and TM polarizations, respectively. Besides the bulk DNR, the surface states therein also possess a nodal point at Γ ($k_x = 0,k_y = 0$) due to the TE and TM degeneracy protected by the rotational symmetry. Since the rotational-symmetry-protected surface nodal point (SNP) is far away from the DNR within the spectrum and always beneath the DNR frequency, the resultant surface states are broadband and hence featured by a bowl-like dispersion. Accordingly, we name this new type of surface state double-bowl state (DBS). Moreover, owing to the fact that the TE and TM polarizations are degenerate at both the DNR and SNP, the surface states are nearly degenerate over the entire spectrum range (Supplementary information Sec. V). This feature is distinct from all previous topological surface states which are pinned by only a single type of topological degeneracy corresponding to either the nodal point or nodal line. Our system thus offers the possibility of generating surface states with arbitrary polarizations. As we know, the realization of a broadband degeneracy of the TE and TM polarized modes remains elusive in all other waveguides such as dielectric waveguides or surface plasmon waveguides, stemming from the fact that the electric and magnetic responses of optical materials are in general different. Therefore, this kind of degeneracy rooted in our system endows us with more freedom to manipulate photons, and it opens a novel avenue for exploring polarized states through the process of light-matter interaction.

Experimentally, the dispersion of the DBS can be identified from the reflection spectra. The experimental and simulation results for both polarizations are provided in Fig. 3c–f. Compared with those for only the PCs (Supplementary information Sec. VI), the reflection spectra demonstrate a global increase due to the presence of the silver layer. Besides that, we can observe the emergence of a new reflection minimum inside the original bulk band gap (bounded by the black dashed lines). These resonance reflection deeps prove the existence of surface states unambiguously. We then numerically calculate the dispersion of the surface states, of which the results are displayed as the black solid lines in Fig. 3c–f. The black solid lines coincide perfectly with the reflection deeps, which further confirms our argument.

## Discussion

In conclusion, we have experimentally demonstrated a new mechanism to realize a photonic type-II DNLS, which has no counterpart in existing electronic DNLSs where electron spin plays the role of polarization. The dispersion around the DNR is obtained through the angle-resolved transmission measurements. When the photonic DNLS is truncated properly and deposited with a silver film on top, the composite system exhibits broadband DBS for both the TE and TM polarizations. The DBS is identified through the deeps in the angle-resolved reflection spectra. Moreover, the DBS is preserved even if the silver film is replaced by another photonic DNLS with a different truncation (see Supplementary information Sec. VII). Our work suggests that photonic topological systems cannot be adequately classified by spinless space groups. On the application side and considering the extreme field concentration due to the surface states, our system can be regarded as an ideal platform for investigating phenomena that require large field enhancement, such as cavity polaritons and nonlinear optics. Additionally, since the DBS for the TE and TM polarizations are almost degenerate over a large spectrum range, this platform exhibits unique advantages in investigating the light-matter interaction between circular polarized photons and spin or valley electrons in a condensed matter system, such as spin polaritons in a microcavity^37^ or valley electrons in MoS_2_^38^.

## Materials and methods

### Experiments

#### Sample fabrication

In our experiments, two sets of PC samples were fabricated with electron beam evaporation on top of a 1.0 mm-thick SiO_2_ substrate. The first sample was used to measure the bulk band dispersion, and the second sample to validate the surface state. For the first sample used, as shown in Fig. 2, 12 unit cells with alternating layers of SiO_2_ (layer A, $d_{{{\mathrm{A}}}} = 388\;{{{\mathrm{nm}}}}$) and Ta_2_O_5_ (layer B, $d_{{{\mathrm{B}}}} = 597\;{{{\mathrm{nm}}}}$) were deposited, with a full layer B at the bottom. The scanning electron microscope image of this sample is shown in Fig. 1a, for which the uncertainty of the thickness is under 10 nm. For the second sample used, as shown in Fig. 3, 12 unit cells were first deposited on the SiO_2_ substrate with a full layer B as the first layer. Then an additional layer B with half the thickness $d_{{{\mathrm{B}}}}/2$ was deposited on the top in order to control the dispersion of the surface states. The geometric parameters of the second PC were $d_{{{\mathrm{A}}}} = 402\;{{{\mathrm{nm}}}}$ and $d_{{{\mathrm{B}}}} = 605\;{{{\mathrm{nm}}}}$. After that, a silver film with a thickness of $\left( {{{{\mathrm{25}}}} \pm {{{\mathrm{5}}}}} \right)$ nm was deposited on top of the PC sample with electron beam evaporation.

#### Spectra measurement

The transmission and reflection spectra were measured at room temperature with an Ideaoptics Instrument PG2000-Pro spectrometer (370–1050 nm) with a wavelength resolution of 0.35 nm. The polarization of the incident wave and the transmission and reflection waves were selected with a polarizer. The measurements were performed as the incident angle varies from $0^{{{\mathrm{o}}}}$ to $60^{{{\mathrm{o}}}}$ at intervals of $0.5^{{{\mathrm{o}}}}$. To increase the accuracy and the stability of our measurements, we set the integration time to 200 ms, and we averaged over five independent measurements for both polarizations.

### Simulations

All the simulations for the transmission and reflection spectra were performed with Lumerical FDTD Solutions, a commercial software based on the Finite-Different Time-Domain Method. A two-dimensional geometry was exploited for which the *z*-axis was chosen as the stacking direction and the *x*-axis represented an arbitrary direction parallel to the surface of layers. Air serves as the background medium. The refractive indexes of SiO_2_ and Ta_2_O_5_ were extracted from the measured data (see Supplementary Data I). The relative permittivity of Ag was from the tabulated reference^39^. Fourier periodic boundary conditions were applied in the *x*-direction and perfectly matched layer conditions were introduced for the *z* termini. A plane-wave source was placed at the top boundary, and it generated polarized waves within the frequency regime of interest (530–630 THz). To achieve angle-resolved transmission and reflection spectra, we swept the incident angle from $0^{{{\mathrm{o}}}}$ to $60^{{{\mathrm{o}}}}$ at intervals of $0.5^{{{\mathrm{o}}}}$ for both the TE and TM polarizations, and used two frequency-domain field and power monitors located far away from the structure to record the spectra. All the simulations were performed at standard temperature and pressure (STP).

## Supplementary information

Double-bowl state in photonic Dirac nodal line semimetal

## References

[1] Qi XL, Zhang SC (2011). Topological insulators and superconductors. Rev. Mod. Phys..

[2] Hasan MZ, Kane CL (2010). *Colloquium*: topological insulators. Rev. Mod. Phys..

[3] Lu L (2013). Weyl points and line nodes in gyroid photonic crystals. Nat. Photonics.

[4] Lu L (2015). Experimental observation of Weyl points. Science.

[5] Ozawa T (2019). Topological photonics. Rev. Mod. Phys..

[6] Ma GC, Xiao M, Chan CT (2019). Topological phases in acoustic and mechanical systems. Nat. Rev. Phys..

[7] Chiu CK (2016). Classification of topological quantum matter with symmetries. Rev. Mod. Phys..

[8] Bradlyn B (2017). Topological quantum chemistry. Nature.

[9] Tang F (2019). Comprehensive search for topological materials using symmetry indicators. Nature.

[10] Vergniory MG (2019). A complete catalogue of high-quality topological materials. Nature.

[11] Zhang TT (2019). Catalogue of topological electronic materials. Nature.

[12] Watanabe H, Lu L (2018). Space group theory of photonic bands. Phys. Rev. Lett..

[13] Xiong ZF (2020). Hidden-symmetry-enforced nexus points of nodal lines in layer-stacked dielectric photonic crystals. Light. Sci. Appl..

[14] Fang C (2015). Topological nodal line semimetals with and without spin-orbital coupling. Phys. Rev. B.

[15] Carter JM (2012). Semimetal and topological insulator in perovskite Iridates. Phys. Rev. B.

[16] Kim Y (2015). Dirac line nodes in inversion-symmetric crystals. Phys. Rev. Lett..

[17] Weng HM (2015). Topological node-line semimetal in three-dimensional graphene networks. Phys. Rev. B.

[18] Mullen K, Uchoa B, Glatzhofer DT (2015). Line of Dirac nodes in hyperhoneycomb lattices. Phys. Rev. Lett..

[19] Liu ZK (2014). Discovery of a three-dimensional topological Dirac semimetal, Na_3_Bi. Science.

[20] Xu SY (2015). Discovery of a Weyl fermion semimetal and topological Fermi arcs. Science.

[21] Burkov AA, Hook MD, Balents L (2011). Topological nodal semimetals. Phys. Rev. B.

[22] Xiao M (2020). Experimental demonstration of acoustic semimetal with topologically charged nodal surface. Sci. Adv..

[23] Belopolski I (2019). Discovery of topological Weyl fermion lines and drumhead surface states in a room temperature magnet. Science.

[24] Yan QH (2018). Experimental discovery of nodal chains. Nat. Phys..

[25] Zhang AM (2016). Interplay of Dirac electrons and magnetism in CaMnBi_2_ and SrMnBi_2_. Nat. Commun..

[26] Rhim JW, Kim YB (2015). Landau level quantization and almost flat modes in three-dimensional semimetals with nodal ring spectra. Phys. Rev. B.

[27] Huh Y, Moon EG, Kim YB (2016). Long-range Coulomb interaction in nodal-ring semimetals. Phys. Rev. B.

[28] Shao YM (2020). Electronic correlations in nodal-line semimetals. Nat. Phys..

[29] Guo QH (2019). Observation of three-dimensional photonic Dirac points and spin-polarized surface arcs. Phys. Rev. Lett..

[30] Cai XX (2020). Symmetry-enforced three-dimensional Dirac phononic crystals. Light. Sci. Appl..

[31] Cheng HB (2020). Discovering topological surface states of Dirac points. Phys. Rev. Lett..

[32] Deng WY (2019). Nodal rings and drumhead surface states in phononic crystals. Nat. Commun..

[33] Qiu HH (2019). Straight nodal lines and waterslide surface states observed in acoustic metacrystals. Phys. Rev. B.

[34] Yang EC (2020). Observation of non-abelian nodal links in photonics. Phys. Rev. Lett..

[35] Xiao M, Zhang ZQ, Chan CT (2014). Surface impedance and bulk band geometric phases in one-dimensional systems. Phys. Rev. X.

[36] Soluyanov AA (2015). Type-II Weyl semimetals. Nature.

[37] Kavokin KV (2004). Quantum theory of spin dynamics of exciton-polaritons in microcavities. Phys. Rev. Lett..

[38] Zeng HL (2012). Valley polarization in MoS_2_ monolayers by optical pumping. Nat. Nanotechnol..

[39] Palik, E. D. *Handbook of Optical Constants of Solids* (Academic Press, 1985).

