# Supplementary material for: Double-bowl state in photonic Dirac nodal line semimetal
Source: Light Sci Appl. 2021 Aug 20;10:170. doi: 10.1038/s41377-021-00614-6 (PMC8379272; doi:10.1038/s41377-021-00614-6)
Supplement: Supplementary file 1 — Double-bowl state in photonic Dirac nodal line semimetal [file 41377_2021_614_MOESM1_ESM.pdf]

Supplementary Information for:

Double-bowl State in photonic Dirac nodal line semimetal

Mengying Hu[1], Ye Zhang[1], Xi Jiang[1], Tong Qiao[1], Qiang Wang[2], Shining Zhu[1], Meng Xiao[3], and Hui Liu[1]

[1]*National Laboratory of Solid State Microstructures, School of Physics, Collaborative Innovation Center of Advanced Microstructures, Nanjing University, Nanjing 210093, China.*

[2]*Division of Physics and Applied Physics, School of Physical and Mathematical Sciences, Nanyang Technological University, Singapore 637371, Singapore.*

[3]*Key Laboratory of Artificial Micro- and Nano-structures of Ministry of Education and School of Physics and Technology, Wuhan University, Wuhan 430072, China.*

Correspondence: Meng Xiao (phmxiao@whu.edu.cn); Hui Liu (liuhui@nju.edu.cn).

Section I: Bands crossing condition for off-normal directions

For the normal direction ($k_x = k_y = 0$), the band structure is given by

$$\cos\left(k_z\Lambda\right) = \cos\left(k_0 n_\mathrm{A} d_\mathrm{A}\right)\cos\left(k_0 n_\mathrm{B} d_\mathrm{B}\right) - \frac{1}{2}\left(\frac{\zeta_\mathrm{A}}{\zeta_\mathrm{B}} + \frac{\zeta_\mathrm{B}}{\zeta_\mathrm{A}}\right)\sin\left(k_0 n_\mathrm{A} d_\mathrm{A}\right)\sin\left(k_0 n_\mathrm{B} d_\mathrm{B}\right) \quad \text{(S1)}$$

where k_z is the Bloch wave vector, Λ is the unit cell length, k_0 is the wave vector in vacuum, $\zeta_i = \sqrt{\mu_i / \varepsilon_i}$, n_i and d_i denote respectively, the impedance, refractive index and the thickness of layer i ($i = \mathrm{A}$ or B). For normal incidence, TE and TM modes exhibit the same band structure. The accidental degeneracies occur at $n_\mathrm{A} d_\mathrm{A} / n_\mathrm{B} d_\mathrm{B} = m_1 / m_2 \in \mathbb{Q}$, where $\{m_1, m_2\} \in \mathbb{N}^+$. As a result, the $(m_1 + m_2)^{\mathrm{th}}$ band and the $(m_1 + m_2 + 1)^{\mathrm{th}}$ band cross at $f_{m_1+m_2} = (m_1 + m_2)c / 2(n_\mathrm{A} d_\mathrm{A} + n_\mathrm{B} d_\mathrm{B})$, where c is the speed of light in vacuum. Note here, the band crossing condition is independent of the impedance and only depends on the ratio of optical path inside each layer. This bands crossing condition for the normal direction have been analytically derived in Ref. [1].

In this section, we show that this kind of crossing condition can be extended to off-normal directions. Since the system is isotropic along in-plane directions, we set $k_y = 0$ for simplicity when we consider off-normal directions. The band structure of a dielectric binary PC with a certain k_x can be written as:

$$\cos\left(k_z\Lambda\right) = \cos\left(k_{z\mathrm{A}} d_\mathrm{A}\right)\cos\left(k_{z\mathrm{B}} d_\mathrm{B}\right) - \frac{1}{2}\left(\frac{\mu_\mathrm{B} k_{z\mathrm{A}}}{\mu_\mathrm{A} k_{z\mathrm{B}}} + \frac{\mu_\mathrm{A} k_{z\mathrm{B}}}{\mu_\mathrm{B} k_{z\mathrm{A}}}\right)\sin\left(k_{z\mathrm{A}} d_\mathrm{A}\right)\sin\left(k_{z\mathrm{B}} d_\mathrm{B}\right) \quad \text{(S2)}$$

for TE polarized mode, and

$$\cos\left(k_z\Lambda\right) = \cos\left(k_{z\mathrm{A}} d_\mathrm{A}\right)\cos\left(k_{z\mathrm{B}} d_\mathrm{B}\right) - \frac{1}{2}\left(\frac{\varepsilon_\mathrm{B} k_{z\mathrm{A}}}{\varepsilon_\mathrm{A} k_{z\mathrm{B}}} + \frac{\varepsilon_\mathrm{A} k_{z\mathrm{B}}}{\varepsilon_\mathrm{B} k_{z\mathrm{A}}}\right)\sin\left(k_{z\mathrm{A}} d_\mathrm{A}\right)\sin\left(k_{z\mathrm{B}} d_\mathrm{B}\right) \quad \text{(S3)}$$

for TM polarized mode. Here, $k_{zi} = \sqrt{k_i^2 - k_x^2}$ ($i = \mathrm{A}$ or B). It is noteworthy that only k_{zi} in Eqs. (S1) and (S2) depends on k_x. For normal incidence, k_{zi} is proportional to n_i, *i.e.*, $k_{zi} = n_i k_0$. As for the off-normal directions, we define an effective refractive index which describes the propagation phase delay along the *z* direction as

$$\tilde{n}_i = \sqrt{\varepsilon_i \mu_i - k_x^2 / k_0^2} \quad \text{(S4)}$$

such that $k_{zi} = \sqrt{k_i^2 - k_x^2} = \tilde{n}_i k_0$. Comparing Eqs. (S2) and (S3) with Eq. (S1), we can see that they

all exhibit the same form if we redefine $\tilde{\zeta}_i^{\mathrm{TE}} \equiv \mu_i / \tilde{n}_i$, $\tilde{\zeta}_i^{\mathrm{TM}} \equiv \tilde{n}_i / \varepsilon_i$ ($i = \mathrm{A}$ or B). Thus the bands crossing condition for off-normal directions in both Eqs. (S2) and (S3) can be obtained by replacing n_i with $\tilde{n}_i$, *i.e.*,

$$\tilde{n}_{\mathrm{A}} d_{\mathrm{A}} / \tilde{n}_{\mathrm{B}} d_{\mathrm{B}} = m_1 / m_2 \in \mathbb{Q}, \tag{S5}$$

Meanwhile, the $(m_1 + m_2)^{\mathrm{th}}$ band and the $(m_1 + m_2 + 1)^{\mathrm{th}}$ band cross at $f_{m_1+m_2} = (m_1 + m_2) c / 2(\tilde{n}_{\mathrm{A}} d_{\mathrm{A}} + \tilde{n}_{\mathrm{B}} d_{\mathrm{B}})$. For the TM modes, the bands also cross at the Brewster angle when $\tilde{\zeta}_i^{\mathrm{TM}} \equiv \mu_i / \tilde{n}_i = 1$.

Above we show that the four-fold degeneracy condition is given in Eq. (S5), and here we proceed to show that Eq. (S5) is robust against material dispersions as well as the variation of geometric parameters. Firstly, one can find that rhs of Eq. (S5) is a constant, and lhs of Eq. (S5) is a continuous function of k_x and system parameters. Note here, k_0 of the degenerate point is related to k_x through $k_x^2 + k_z^2 = k_0^2$. Meanwhile, Eq. (S5) is satisfied at isolated k_xs, thus the only possibility is that the lhs as a continuous function of k_x crosses rhs, a constant once at the band degeneracy. In the presence of a small change of the system parameters such as dispersions or thickness, Equation (S5) can still be satisfied with a shift of k_x. As an example, we consider the case where the thickness of two layers change as $d'_{\mathrm{A}} = (1+\alpha) d_{\mathrm{A}}$ and $d'_{\mathrm{B}} = (1+\alpha) d_{\mathrm{B}}$ with α being a small number. Here $d_{\mathrm{A}} = 388\mathrm{nm}$ and $d_{\mathrm{B}} = 597\mathrm{nm}$ are the thickness of the SiO_2 and Ta_2O_5 layers, respectively. The presence of α describes a common system error in fabricating layered structures. Same as the main text, we focus on the four-fold degeneracy point at 591THz. The locations of the four-fold degeneracy point as a function of α is shown in Fig. S1. From Fig. S1, we can see that the four-fold degeneracy point preserves and shifts in k_x as we vary α. SiO_2 and Ta_2O_5 are almost dispersionless within the frequencies of interest. (see Supplementary Data I) Nevertheless, we want to mention that the above argument also works for dispersive material. The four-fold degeneracies preserve and the dispersion of material only introduce a small shift of k_x, similar as the effect of α discussed above.

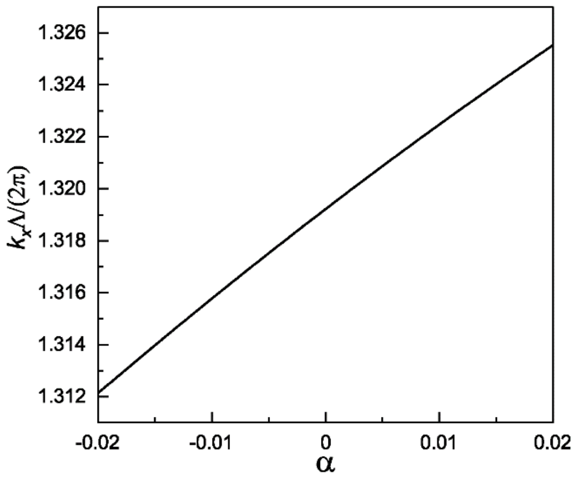

Fig. S1 k_x of the four-fold degeneracy point as a function of α.

Since this system exhibits time reversal symmetry and is in-plane isotropic, the band dispersion should be symmetric for k_z with respect to $k_z = 0$ at an arbitrary fixed k_x. Then we can see that the band dispersions should be monotonic as functions of k_z for $k_z > 0$ or $k_z < 0$ at that fixed k_x, otherwise there will be at least four k_zs (two for $k_z > 0$ and two for $k_z < 0$) for one frequency in the non-monotonic region of the band dispersion. This is contradictory to Eqs. (S2) and (S3) since for each frequency, the rhs of Eqs. (S2) or (S3) is single valued and hence we have at most two k_zs (one positive and one negative) on the lhs. Thus the monotonicity along k_z further leads to the conclusion that the degenerate points of two bands occur only at the zone boundary ($k_z = \pi/\Lambda$) or the zone center ($k_z = 0$).

Section II: Existence of Dirac nodal ring at $k_z = 0$

In the main text, we analyze a Dirac nodal ring (DNR) at $k_z = \pi/\Lambda$ whose location is shown with the golden ring in Fig. 1e. Here in this section, we provide the band dispersion for another DNR at $k_z = 0$. Here the parameters we use are the same as Fig. 1 in the main text, and the DNR corresponds to the orange circle at a lower frequency (around 400THz) in Fig. 1b. Figs. S2a and S2b show the band dispersion along the k_x and k_z direction around the four-fold degeneracy point. Considering

the fact that the system are rotational invariant for in-plane directions, we thus get a DNR located at $k_z = 0$ as sketched in Fig. S2c.

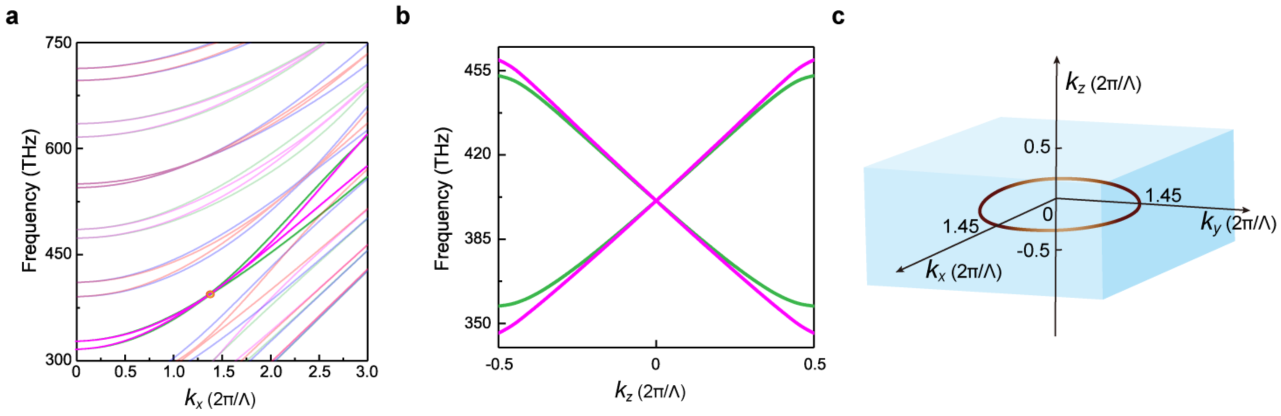

Fig. S2 Band dispersion along **a** k_x and **b** k_z for a DNR at $k_z = 0$ (green and magenta). The magenta and blue (olive and red) lines represent the band dispersion for TE (TM) polarization. **c**. Sketch of the Dirac nodal ring in the momentum space.

Section III: Transmission spectra at different φs

To verify the in-plane isotropy of our system, we measured transmission spectra at various azimuthal angles φs besides $\varphi = 0^{\circ}$ shown in the main text. Fig. S3 displays the transmission spectra for both TE and TM polarizations at $\varphi = 0^{\circ}$, $\varphi = 45^{\circ}$ and $\varphi = 90^{\circ}$, which are almost the same, sufficiently evidencing the isotropy inherent in our system.

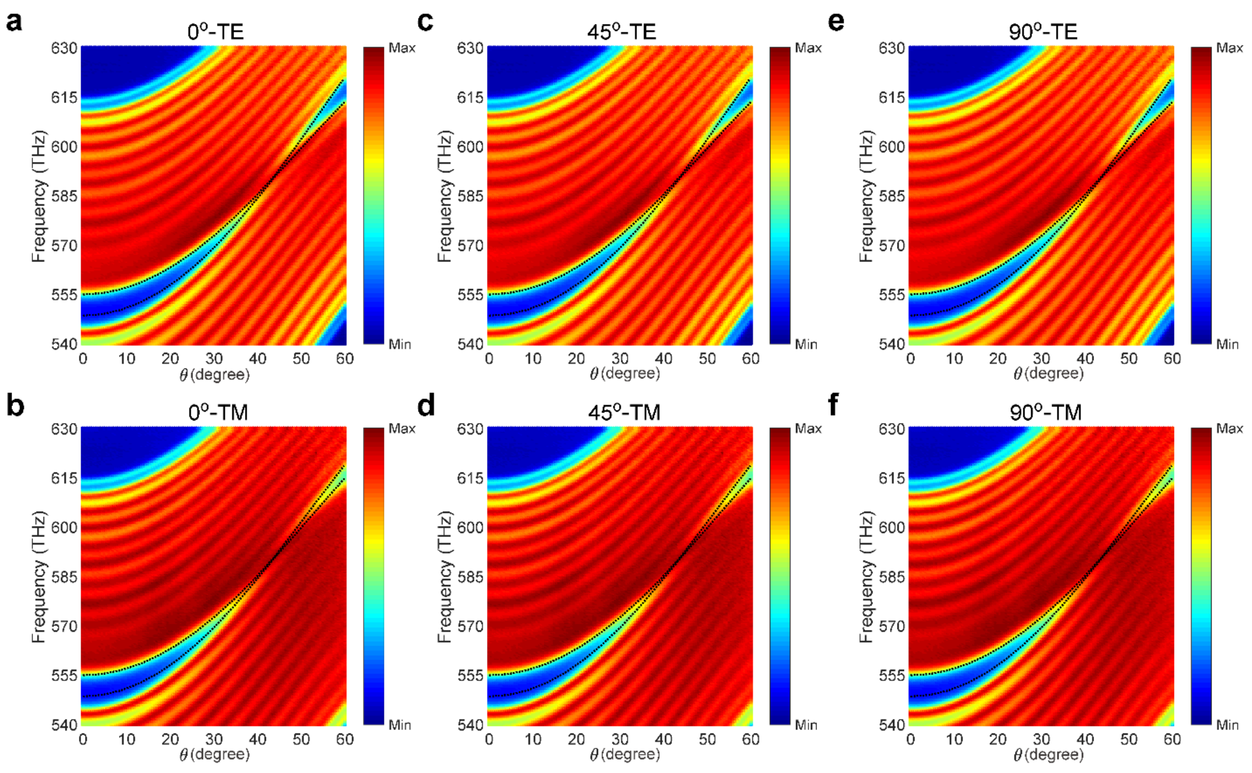

Fig. S3 Measured transmission spectra for both polarizations as **a, b**, $\varphi = 0^{\mathrm{o}}$ **c, d** $\varphi = 45^{\mathrm{o}}$ and **e, f** $\varphi = 90^{\mathrm{o}}$. Black dashed lines mark the band dispersion at $k_z = \pi/\Lambda$.

Section IV: Band dispersion extracted from the Fabry-Perot interference

In this section, we offer details about the approach to extract the band dispersion at the DNR along the k_z direction as shown in Fig. 2. To obtain the dispersion along the k_z direction at the DNR, we need the transmission and reflection spectra at fixed $k_{\rho D} = 1.32(2\pi/\Lambda)$. However, the experimental data are measured at fixed incident angle, thus we first smoothly interpolate the measured spectrum, and then extract the transmission and reflection spectra at fixed $k_{\rho D}$, and the data are shown in Fig. S4a.

The geometry of our system is shown in Fig. S4b, with the number of unit cell N=12. The Fabry-Perot interference pattern for a fixed k_z actually comes from the interference of multiple Bloch modes. Following a similar transfer matrix method used in Ref. [2], we obtain the reflection

and transmission coefficients of the system as

$$R=\left|\frac{(\chi_1-1)\sin\left[(N-1)k_z\Lambda\right]-\left(\chi_1(\chi_2+\chi_3)-\chi_2+\chi_3\right)\sin(Nk_z\Lambda)}{(\chi_1+1)\sin\left[(N-1)k_z\Lambda\right]-\left(\chi_1(\chi_2+\chi_3)+\chi_2-\chi_3\right)\sin(Nk_z\Lambda)}\right|^2 \tag{S6}$$

and

$$T=1-R, \tag{S7}$$

with

$$\begin{cases}\chi_2^{\mathrm{TE}}=\dfrac{k_{z0}}{k_{z\mathrm{A}}}\\ \chi_2^{\mathrm{TE}}=e^{ik_{z\mathrm{A}}d_\mathrm{A}}\left(\cos(k_{z\mathrm{B}}d_\mathrm{B})+\dfrac{i}{2}\left(\dfrac{\tilde{\zeta}_\mathrm{A}^{\mathrm{TE}}}{\tilde{\zeta}_\mathrm{B}^{\mathrm{TE}}}+\dfrac{\tilde{\zeta}_\mathrm{B}^{\mathrm{TE}}}{\tilde{\zeta}_\mathrm{A}^{\mathrm{TE}}}\right)\sin(k_{z\mathrm{B}}d_\mathrm{B})\right)\\ \chi_3^{\mathrm{TE}}=e^{ik_{z\mathrm{A}}d_\mathrm{A}}\left(-\dfrac{i}{2}\left(\dfrac{\tilde{\zeta}_\mathrm{A}^{\mathrm{TE}}}{\tilde{\zeta}_\mathrm{B}^{\mathrm{TE}}}-\dfrac{\tilde{\zeta}_\mathrm{B}^{\mathrm{TE}}}{\tilde{\zeta}_\mathrm{A}^{\mathrm{TE}}}\right)\sin(k_{z\mathrm{B}}d_\mathrm{B})\right)\end{cases} \tag{S8}$$

and

$$\begin{cases}\chi_2^{\mathrm{TM}}=\dfrac{n_\mathrm{A}^2k_{z0}}{n_0^2k_{z\mathrm{A}}}\\ \chi_2^{\mathrm{TM}}=e^{ik_{z\mathrm{A}}d_\mathrm{A}}\left(\cos(k_{z\mathrm{B}}d_\mathrm{B})+\dfrac{i}{2}\left(\dfrac{\tilde{\zeta}_\mathrm{A}^{\mathrm{TM}}}{\tilde{\zeta}_\mathrm{B}^{\mathrm{TM}}}+\dfrac{\tilde{\zeta}_\mathrm{B}^{\mathrm{TM}}}{\tilde{\zeta}_\mathrm{A}^{\mathrm{TM}}}\right)\sin(k_{z\mathrm{B}}d_\mathrm{B})\right)\\ \chi_3^{\mathrm{TM}}=e^{ik_{z\mathrm{A}}d_\mathrm{A}}\left(-\dfrac{i}{2}\left(\dfrac{\tilde{\zeta}_\mathrm{A}^{\mathrm{TM}}}{\tilde{\zeta}_\mathrm{B}^{\mathrm{TM}}}-\dfrac{\tilde{\zeta}_\mathrm{B}^{\mathrm{TM}}}{\tilde{\zeta}_\mathrm{A}^{\mathrm{TM}}}\right)\sin(k_{z\mathrm{B}}d_\mathrm{B})\right)\end{cases} \tag{S9}$$

for TE and TM polarizations, respectively. Here k_{z0} is the *z*-component of the wave vector in the air. The comparison between these analytical results (solid lines) and numerical simulations (open circles) are provided in Fig. S4c. In Eq. S7 (S6), the transmission peak (reflection deep) is reached when

$$\sin(Nk_z\Lambda)=0 \tag{S10}$$

or

$$Nk_z\Lambda=m\pi, \tag{S11}$$

where *m* is an integer. Under such a condition, the transmission maximum is simplified as

$$T_{\max}=\frac{4\chi_1}{(1+\chi_1)^2} \tag{S12}$$

$T_{\max}$ as functions of frequency are for both polarization also provided as dashed lines in Fig. S4c for comparison. Equation (S11) builds a relation between the values of Bloch wave vector k_z and the corresponding frequencies at which T (R) reaches the peak (deep) value, and which then gives the band dispersion we extract and provided in Figs. 2e and 2f in the main text.

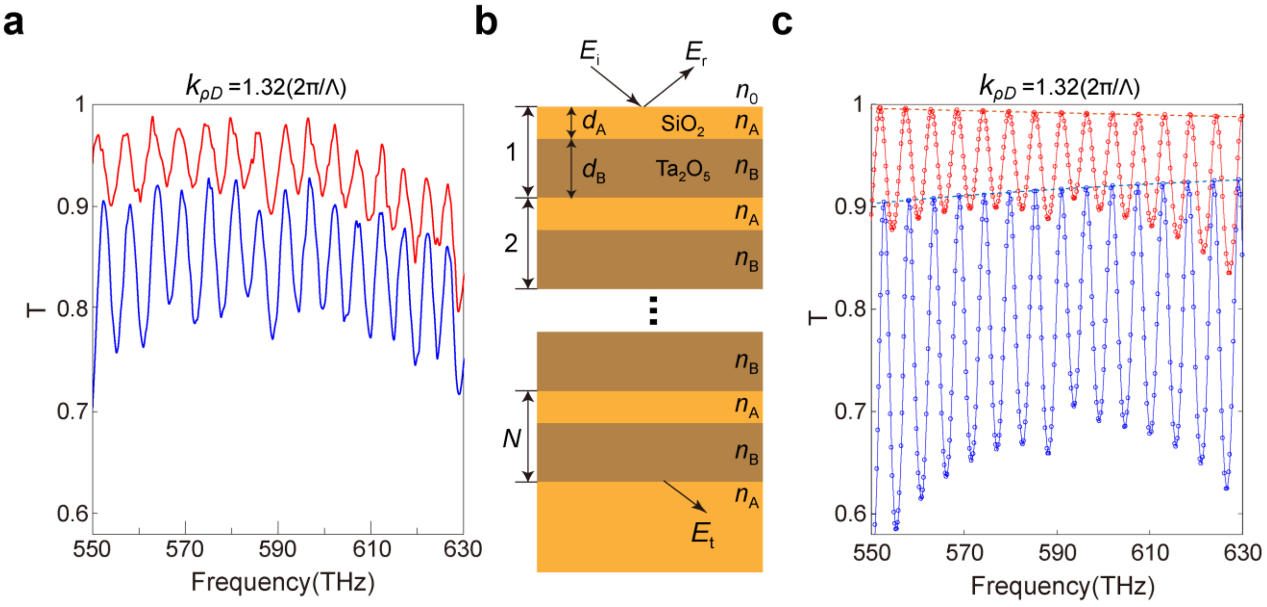

Fig. S4 **a.** Measured transmission spectra for TE (blue) and TM (red) polarizations at $k_{\rho D} = 1.32(2\pi/\Lambda)$. **b.** Sketch of our system. The plane wave incidents on the sample from air ($n_0 = 1$) and transmit into the substrate made of SiO2. The incident, reflected and transmitted amplitudes of electric field are labeled as E_{i}, E_{r} and E_{t}, respectively. **c.** Analytical (solid lines) and numerical (open circles) results of transmission spectra at $k_{\rho D} = 1.32(2\pi/\Lambda)$, together with the functions $T_{\max}$ (dashed lines) for TE (blue) and TM (red) polarizations.

Section V: Nearly degeneracy of the double-bowl surface states

A double-bowl surface state consists of two drumhead surface states for TE and TM polarizations, and these two drumhead surface states are degenerate at Γ and DNR. Actually, these two drumhead surface states are almost degenerate over the whole spectra range as revealed in Fig. S5, where we

render the trajectories in $k_x - f$ plane for three sets of drumhead surface states corresponding to those in Figs. 3a and 3b. Meanwhile, such a nearly ideal degeneracy can be extended all over the spectrum by tuning $n_A d_A / n_B d_B$, which is experimentally feasible. Therefore, our scheme holds great potential for generating drumhead surface states with arbitrary polarizations.

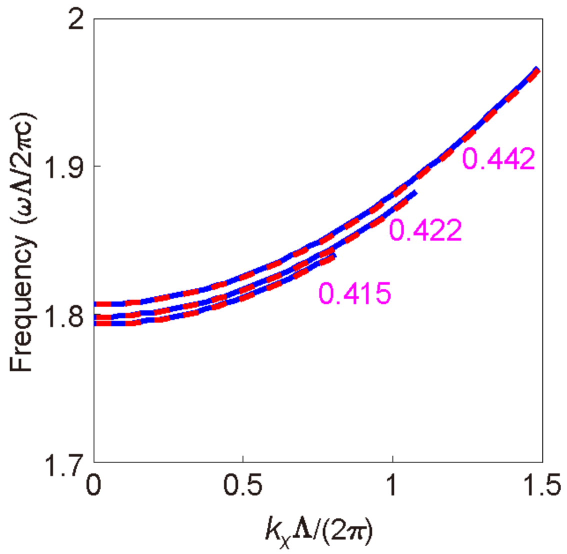

Fig. S5 Drumhead surface states with TE (blue solid lines) and TM (red dashed lines) polarizations as $n_A d_A / n_B d_B$ from top to bottom equals 0.442, 0.422 and 0.415, respectively. The sum $n_A d_A + n_B d_B$ remains constant in these three cases.

Moreover, the surface states can either be expanded by the DNR or extended from the DNR to infinity depending on the detail of the PC surface truncation. For the case shown in the main text, the PC is truncated with layer B of thickness $d_B/2$ on top, wherein the composite system exhibits drumhead surface states for both TE and TM polarizations. Here, we fabricate another PC sample truncated with a complete layer A (other parameters are identical to the sample used in Fig. 2), and on top of which we deposit a 25nm silver film. With the aid of angle-resolved transmission measurements, we are capable to achieve surface states extended from the DNR to infinity, which display themselves as transmission peaks inside the original bulk band gap (bounded by the black dashed lines) in Fig. S6.

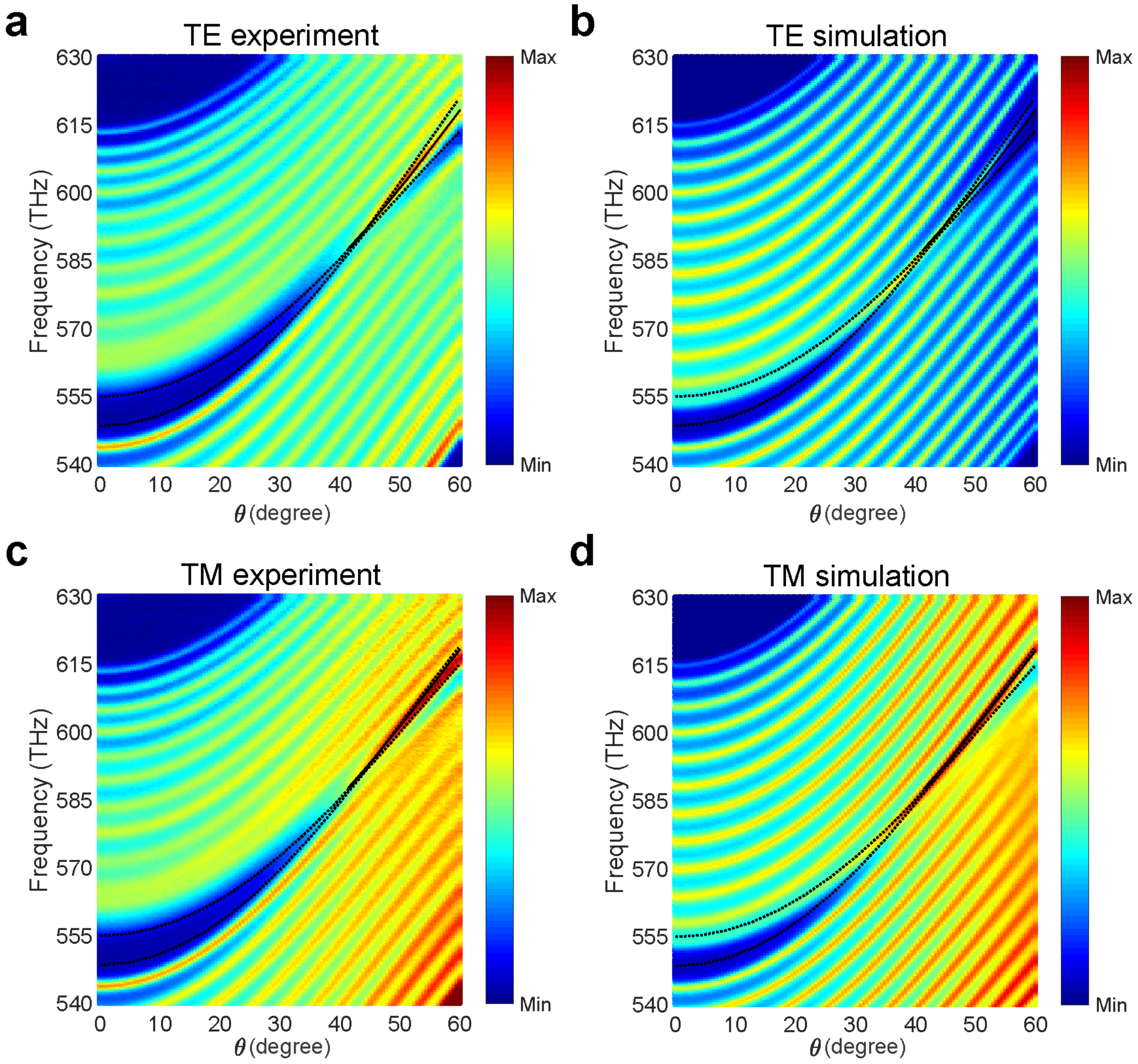

Fig. S6 Surface states under oblique-incident excitations. **a, c**, measured and **b, d**, simulated transmission spectra for **a, b**, TE and **c, d**, TM excitations, in which the surface states extend from the DNR to infinity. Black solid lines exhibit eigenfrequencies of the surface states, while black dashed lines mark the boundary of the projected band regions.

Section VI: Reflection spectra of the PC with/without a silver film

Here in this section we provide the reflection spectra of the PC before deposited with a silver film. The parameters of the PC are the same as that used in Fig. 3. Here Figs. S7a and S7b (Figs. S7c and S7d) show the reflection spectra without (with) the silver film for the TE and TM polarization,

respectively. It can be seen that, when deposited with a silver film, there is a global increase of the reflection spectra. Note here for clearance, we show the absolute values of the reflectance. Meanwhile, there is an additional reflection deep emerging inside the original band gap which corresponds to the double-bowl states.

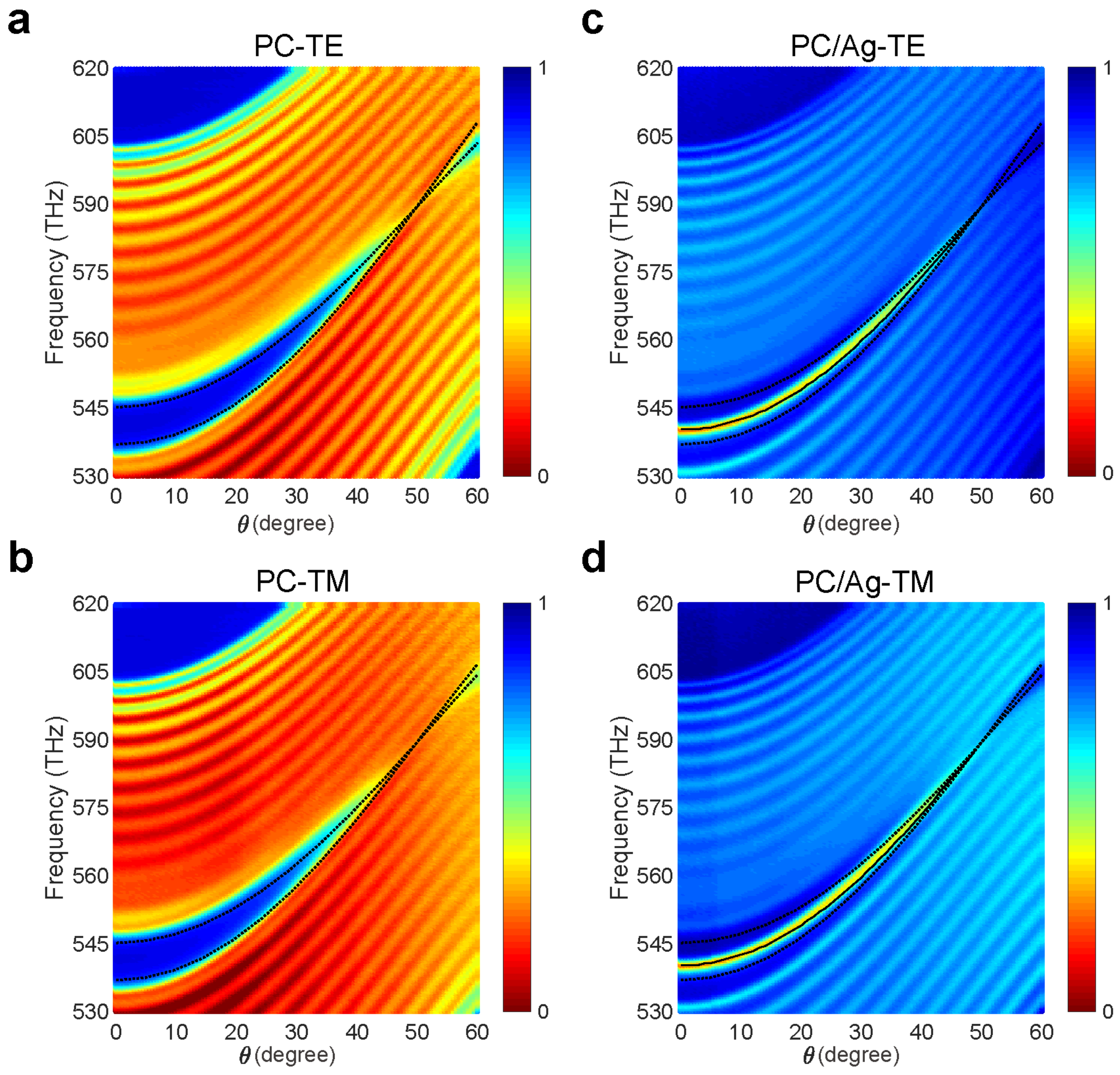

Fig. S7 Reflection spectra for the PC without (**a**, **b**) and with (**c**, **d**) a silver film of 25nm deposited on top. The PC studied here is the same as the PC in Fig. 3 of the main text. Black dashed lines correspond to the boundaries of projected bands, and the black solid line in **c** and **d** mark the dispersion of the double-bowl states.

Section VII: Double-bowl surface states between two photonic DNLSs

In this section we show that the double-bowl surface states can also be supported by interface between two photonic DNLSs. Here these two photonic DNLSs are the same only with different truncations at the interface. As shown in Fig. S8a, the silver film is replaced with another photonic DNLS whose first layer is SiO_2 with half the thickness $d_A/2$. Besides that, we also assume the two photonic DNLS are semi-infinite. The projected band together with the double-bowl surface states are shown in Fig. S8b, where the blue (red) region depicts the projected band of TE (TM) polarization, and the lines represent the surface states localized between two photonic DNLS. It is intriguing to see that such a configuration exhibits two sets of surface states: one is the double-bowl surface states and the other surface states extend from the DNR to infinity.

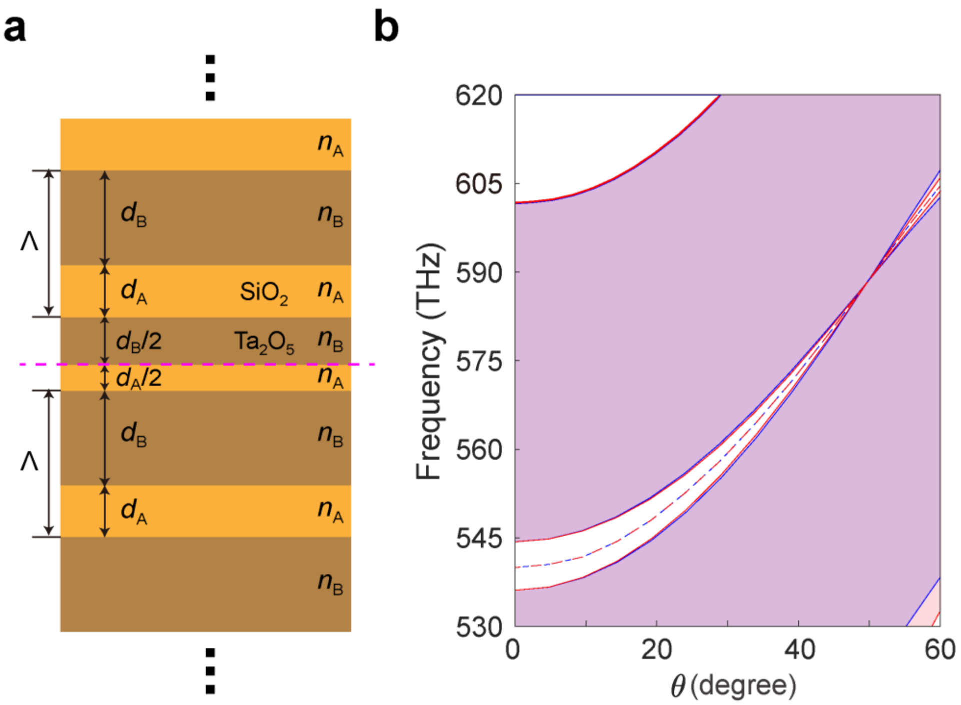

Fig. S8 **a**. Interface formed between two same photonic DNLS with different truncations. **b**. Projected band structure and the surface state dispersion. Here the red and blue regions represent the projected band of TE and TM polarizations, respectively, and the blue (red) dashed line is the dispersion of the surface states with TE (TM) polarization. Here, the thickness of the SiO_2 layer and Ta_2O_5 layer are $d_A = 402\text{nm}$ and $d_B = 605\text{nm}$, respectively, and the refractive indexes are provided in Supplementary Data I.

Supplementary Data I: Refractive indexes of SiO_2 and Ta_2O_5

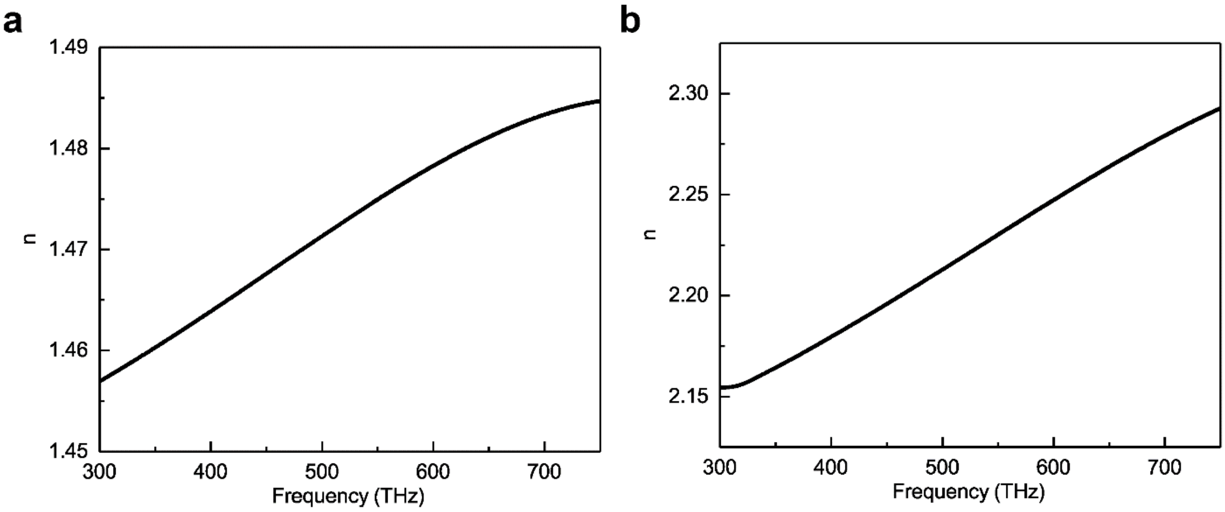

Fig. S9 Measured refractive indexes (*n*) of SiO_2 (**a**) and Ta_2O_5 (**b**) for the frequencies of interest. SiO_2 and Ta_2O_5 are nonmagnetic material with relative permeability both equal to 1.

Supplementary References

1 Xiao, M., Zhang, Z. Q. & Chan, C. T. Surface impedance and bulk band geometric phases in one-dimensional systems. *Physical Review X* **4**, 021017 (2014).

2 Yariv, A. & Yeh, P. Photonics: Optical Electronics in Modern Communications Ch. 12 (Oxford Univ. Press, Oxford, 2007).
